# Fish female-biased gene *cyp19a1a* leads to female antiviral response attenuation between sexes by autophagic degradation of MITA

**DOI:** 10.1371/journal.ppat.1010626

**Published:** 2022-06-21

**Authors:** Long-Feng Lu, Jing-Yu Jiang, Wen-Xuan Du, Xue-Li Wang, Zhuo-Cong Li, Xiao-Yu Zhou, Can Zhang, Cheng-Yan Mou, Dan-Dan Chen, Zhi Li, Li Zhou, Jian-Fang Gui, Xi-Yin Li, Shun Li

**Affiliations:** 1 Institute of Hydrobiology, Chinese Academy of Sciences, Wuhan, China; 2 University of Chinese Academy of Sciences, Beijing, China; 3 College of Fisheries and Life Science, Dalian Ocean University, Dalian, China; 4 State Key Laboratory of Freshwater Ecology and Biotechnology, Hubei Hongshan Laboratory, The Innovative Academy of Seed Design, Chinese Academy of Sciences, Wuhan, China; University of Pennsylvania, UNITED STATES

## Abstract

From insects to mammals, both innate and adaptive immune response are usually higher in females than in males, with the sex chromosome and hormonal differences considered the main reasons. Here, we report that zebrafish *cyp19a1a* (cytochrome P450, family 19, subfamily A, polypeptide 1a), an autosomal gene with female-biased expression, causes female fish to exhibit a lower antiviral response. First, we successfully constructed an infection model by intraperitoneal injection of spring viremia of carp virus (SVCV) into zebrafish (*Danio rerio*) and *Carassius auratus* herpesvirus (*Ca*HV) in gibel carp (*Carassius gibelio*). Specifically, female fish were more vulnerable to viral infection than males, accompanied by a significantly weaker interferon (IFN) expression. After screening several candidates, *cyp19a1a*, which was highly expressed in female fish tissues, was selected for further analysis. The IFN expression and antiviral response were significantly higher in *cyp19a1a*^-/-^ than in *cyp19a1a*^+/+^. Further investigation of the molecular mechanism revealed that Cyp19a1a targets mediator of IRF3 activation (MITA) for autophagic degradation. Interestingly, in the absence of MITA, Cyp19a1a alone could not elicit an autophagic response. Furthermore, the autophagy factor ATG14 (autophagy-related 14) was found interacted with Cyp19a1a to either promote or attenuate Cyp19a1a-mediated MITA degradation by either being overexpressed or knocked down, respectively. At the cellular level, both the normal and MITA-enhanced cellular antiviral responses were diminished by Cyp19a1a. These findings demonstrated a sex difference in the antiviral response based on a regulation mechanism controlled by a female-biased gene besides sex chromosome and hormonal differences, supplying the current understanding of sex differences in fish.

## Author summary

Sex chromosome and hormonal differences are considered as the crucial mechanisms of sexual difference in antiviral response. In this study, we report that the fish autosomal gene with female-biased expression *cyp19a1a*, leads to a weaker antiviral response in female fish. First, female fish were vulnerable than males when infected with virus, and with a lower IFN expression. *cyp19a1a* was evoked by viral stimulation and highly expressed in female fish tissues. In *cyp19a1a*^-/-^, the IFN response and antiviral capacity were notably higher than that in *cyp19a1a*^+/+^. Subsequently, Cyp19a1a was interacted with MITA for autophagic degradation, and Cyp19a1a could not lead to an autophagic response in the absence of MITA, in this process, ATG14 was identified associated with Cyp19a1a to promote MITA degradation. These results demonstrated that the autosomal and female-biased gene *Cyp19a1a* was an IFN negative regulator by mediating MITA autophagic degradation, extending the understanding of sex differences in vertebrates besides sex chromosome and hormonal differences.

## Introduction

Sex is prevalent among eukaryotes and females and males commonly display significant sexual differences in morphology, physiology, and behavior [1–3]. Sex-based immune response differences have been reported in several species, in which females usually exhibit stronger immunity responses [4]. Such responses in the host includes hypersensitivity, release of pro-inflammatory cytokines, and antibody (Ab) responses. According to previous reports, sex chromosomes and hormonal levels are crucially involved in the immune response difference between sexes. For example, the encoded G protein coupled receptor GPR174 on the X chromosome inhibits germinal center formation in males and not females, and is considered a major reason for generally lower ab-mediated autoimmunity in males [5]. Furthermore, estrogen receptors predominantly exist in B and T lymphocytes; females generate higher levels of estrogen, hormonal difference that leads to a stronger humoral immune response [6,7]. Differences in susceptibility to viral infections have also been observed between males and females. Compared to that in males, female plasmacytoid dendritic cells produce higher type I interferon (IFN) during human immunodeficiency virus (HIV)-1 infection and lesser HIV RNA is detected in women, which increases slower to a higher viral load level [8]. During viral infection, sexually dimorphic phenotypes are almost entirely focused on immune cell numbers and function. In fact, other than the sex chromosome and hormonal differences, there are other indicators to distinguish between males and females, such as the specifically expressed sex-related genes. However, the functions of these genes in immune response differences between sexes have not been elucidated.

Cyp19 is an enzyme found in all vertebrates that is crucially involved in gonadal maturation and shown to catalyze the conversion of androgens to estrogens [9]. Mammals only have a single *cyp19* gene; in contrast, most teleosts such as zebrafish possess two structurally and functionally distinct *cyp19* genes: *cyp19a1a* encodes aromatase A and *cyp19a1b* encodes aromatase B [10,11]. It has been reported that Cyp19a1a is crucially involved in sexual and gonadal differentiation in zebrafish; knocking out zebrafish *cyp19a1a* has been shown to lead to all-male offspring [12–14]. Although the vital function of Cyp19a1a in sexual development has been extensively investigated to date, its involvement in the host immune response, in particular, its role in IFN expression is still unknown.

The first line of defense against viral infections is the innate immune response that depends on the cellular pattern recognition receptors (PRRs) and sense conserved pathogen-associated molecular patterns (PAMPs) [15,16]. Virus-derived nucleic acids are mainly detected by DNA and RNA sensors such as retinoic acid-inducible gene I (RIG-I)-like receptors (RLRs), including RIG-I, melanoma differentiation-associated factor 5 (MDA5), and laboratory of genetics and physiology 2 (LGP2) [17,18]. The RLR signaling pathway is among the major pathways involved in the production of active IFN and other cytokines [19,20]. Numerous studies have reported that the mediator of IFN regulatory factor 3 (IRF3) activation (MITA), part of the RLR cascade, is an important mediator of innate immune response [21]. Upon viral infection, MITA undergoes phosphorylation, ubiquitination, and subcellular translocation to subsequently function as an adaptor protein that recruits TANK-binding kinase 1 (TBK1) and IRF3 to the mitochondrial antiviral signaling protein (MAVS)-associated complex, thereby facilitating the activation of IRF3 [22]. Controlling the MITA expression in the host effectively regulates IFN expression. Negative regulation events of MITA include: E3 ubiquitin ligase RING finger protein 5 (RNF5) catalyzes the K48-linked ubiquitination of MITA and promotes its proteasome-dependent degradation [23]; Ubiquitin-specific protease 13 (USP13) deconjugates the K27-linked polyubiquitin chains from MITA and prevents the recruitment of TBK1 to MITA [24]. Recently, a few studies have demonstrated that fish MITA is modulated by several factors to regulate IFN expression. For instance, zebrafish IRF10 has been shown to inhibit IFN activation by blocking MITA activation [25]. Although MITA is pivotal for IFN activation in fish, its regulatory mechanisms remain unelucidated.

In this study, Cyp19a1a is involved in the sex-based immune response difference in fish. Higher mortality was observed in female fish upon viral infection. The expression of the antiviral factor IFN was found lower in female. After screening sex-biased genes, *cyp19a1a* was identified to have a potential role in affecting immune response differences between the sexes. Knocking out *cyp19a1a* in zebrafish significantly enhanced their antiviral response capacity. Further analyses demonstrated that Cyp19a1a promotes the autophagic degradation of MITA to suppress IFN expression. These findings revealed that the female-biased molecule, Cyp19a1a, decreases the IFN activation and antiviral response capacity in female fish by targeting MITA.

## Results

### Female fish are more vulnerable to viral infection than male fish

**Fig 1:**
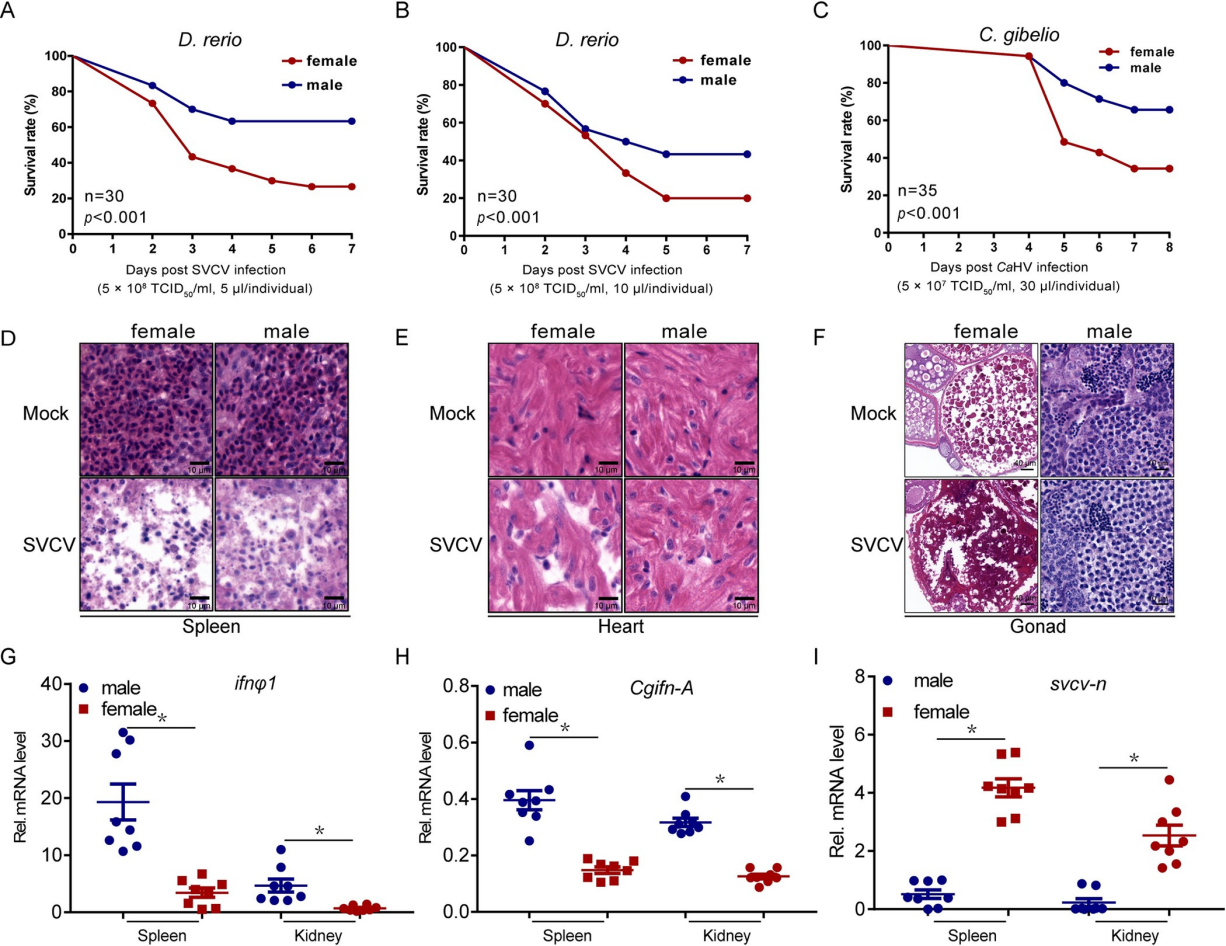
Sex-dependent fish susceptibility to mortality by SVCV infection. (A-B) Survival (Kaplan-Meier Curve) of male and female zebrafish (n = 30 per group) at various days after i.p. injected with SVCV (5 × 10^8^ TCID_50_/ml, 5 μl or 10 μl/individual). (C) Wild-type Male and female gibel carp (n = 35 per group) were i.p. injected with *Ca*HV (5 × 10^7^ TCID_50_/ml, 30 μl/individual) and the survival rate was calculated. (D-F) Microscopy of H&E-stained spleen (D), heart (E), and gonad (F) sections from male and female zebrafish treated with SVCV (5 × 10^8^ TCID_50_/ml, 5 μl/individual) for 72 h. (G) qPCR analysis of *ifnφ1* mRNA in the spleen and kidney of male and female zebrafish (n = 8 per group) given i.p. injection of SVCV (5 × 10^8^ TCID_50_/ml, 5 μl/individual) for 48 h. Each dot point represents one independent biological replicate. *p < 0.05. (H) qPCR analysis of *Cgifn-A* mRNA in the spleen and kidney of male and female gibel carp (n = 8 per group) given i.p. injection of *Ca*HV (5 × 10^7^ TCID_50_/ml, 30 μl/individual) for 48 h. Each dot point represents one independent biological replicate. *p < 0.05. (I) qPCR analysis of *svcv-n* mRNA in the spleen and kidney of zebrafish (n = 8 per group) given i.p. injection of SVCV (5 × 10^8^ TCID_50_/ml, 5 μl/individual) for 48 h. *p < 0.05.

First, differences in antiviral response capacity of fish based on sex were investigated by intraperitoneally (i.p.) injecting age-matched female and male zebrafish or gibel carp with spring viremia of carp virus (SVCV) or *Carassius auratus* herpesvirus (*Ca*HV). Within 7 days, significantly higher mortality was observed in female fish compared to that in male fish; a similar higher mortality was also observed in female zebrafish upon infection with high viral titers (Fig 1A–1C). Consistent with this decreased survival rate, increased tissue damage was observed in the spleen, heart, and gonad after viral infection (Fig 1D–1F). In order to verify the antiviral response difference between female and male fish, the expression levels of the vital factor, type I IFN, were determined in several tissue types. There are four type I IFN genes in zebrafish, only IFNφ1 and IFNφ3 are induced by polyinosinic:polycytidylic acid (poly I:C), the mimic of viral RNA, and IFNφ3 could not be activated by IRF3 which unlike IFNφ1, therefore, IFNφ1 was chosen to represent the zebrafish IFN [26]. Consistent with the histopathologic results, the expression patterns of *ifnφ1* in tissues were also distinct between the sexes. Females showed notably lower production of *ifnφ1* or *Cgifn-A* in the immune organs, spleen and kidney, and higher viral gene transcription, such as the *svcv-n* gene transcription, compared to males (Fig 1G–1I). Collectively, these results demonstrated the weaker antiviral response capacity in female fish than in male fish.

### Higher IFN activation and stronger antiviral response in *cyp19a1a* deficient zebrafish

**Fig 2:**
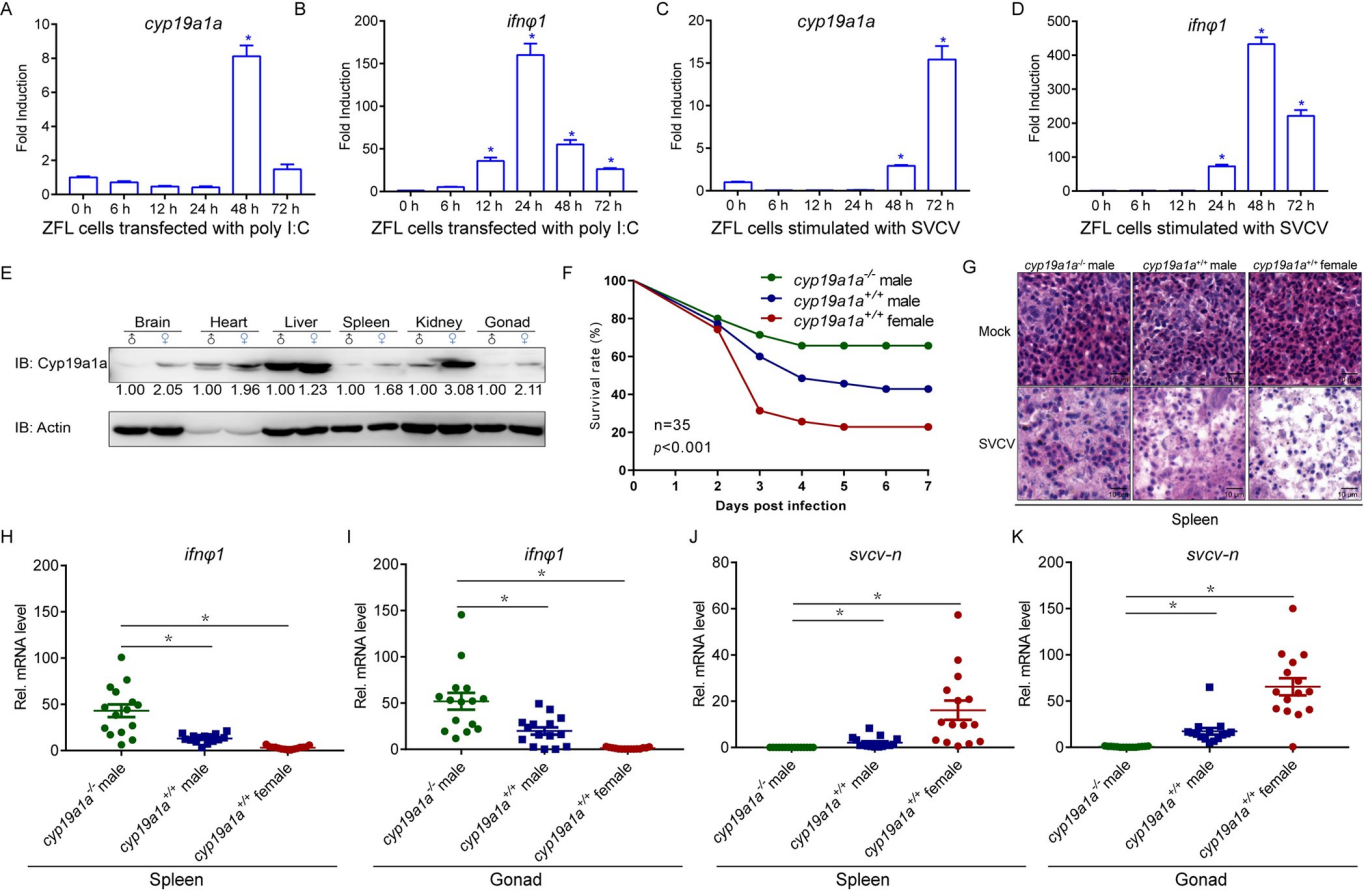
Loss of *cyp19a1a* protect zebrafish from virus infection. (A-D) *Cyp19a1a* is induced by virus infection. qPCR detection of the mRNA levels of *cyp19a1a and ifnφ1* on stimulation. ZFL cells were seeded in 3-cm^2^ dishes overnight and transfected with poly I:C (1 μg/ml) (A and B) or infected with SVCV (MOI = 1) (C and D). At the time points 6, 12, 24, 48, and 72 h, total RNAs were extracted for further qPCR assays. The relative transcriptional levels were normalized to the transcription of *β-actin* and represented as fold induction relative to the transcriptional level in control cells, which was set to 1. Data were expressed as mean ± SEM, *n* = 3. Asterisks indicate significant differences from control (*, *p* < 0.05). (E) IB analysis of endogenous Cyp19a1a protein level in the brain, heart, liver, spleen, kidney, and gonad of male and female zebrafish (n = 3 per group). (F) Survival (Kaplan-Meier Curve) of *cyp19a1a*^*-/-*^ male, *cyp19a1a*^*+/+*^ male, and *cyp19a1a*^*+/+*^ female zebrafish (n = 35 per group) at various days after i.p. injected with SVCV (5 × 10^8^ TCID50/ml, 5 μl/individual). (G) Microscopy of H&E-stained spleen section from *cyp19a1a*^*-/-*^ male, *cyp19a1a*^*+/+*^ male, and *cyp19a1a*^*+/+*^ female zebrafish treated with SVCV (5 × 10^8^ TCID_50_/ml, 5 μl/individual) for 72 h. (H-K) qPCR analysis of *ifnφ1* and *svcv-n* mRNA in the spleen and gonad of *cyp19a1a*^*-/-*^ male, *cyp19a1a*^*+/+*^ male, and *cyp19a1a*^*+/+*^ female zebrafish (n = 15 per group) given i.p. injection of SVCV (5 × 10^8^ TCID50/ml, 5 μl/individual) for 48 h. Each dot point represents one independent biological replicate. *p < 0.05.

After screening and analyzing several sex-biased genes, the *cyp19a1a* gene highly expressed in the female fish was selected for further investigation (S1 Fig). To identify whether *cyp19a1a* is related to the antiviral response, ZFL cells were stimulated with poly I:C or virus. The result suggested that *cyp19a1a* was significantly upregulated, indicating that it was involved in the antiviral process (Fig 2A–2D). As previously reported in other studies, Cyp19a1a were confirmed to be primarily expressed in the female (Fig 2E). Cyp19a1a is an essential factor for ovary generation during fish development; therefore, only male *cyp19a1a*^-/-^ zebrafish could be obtained, which limited the experiment group. The male *cyp19a1a*^-/-^ mutants exhibited higher survival rates compared to those of *cyp19a1a*^+/+^ wild type male and female fish after viral infection (Fig 2F). A lesser degree of spleen tissue damage was observed in *cyp19a1a*^-/-^ group, comparing with in both wild type group (Fig 2G). Moreover, *cyp19a1a*^-/-^ mutants also exhibited high levels of *ifnφ1* in the spleen and gonad in response to SVCV infection (Fig 2H and 2I). Consistent with these observations, lower *svcv-n* gene expressions were detected in *cyp19a1a*^-/-^ zebrafish (Fig 2J and 2K). These results suggested that *cyp19a1a*^-/-^ zebrafish exhibited stronger antiviral response, accompanied with higher IFN production.

### Cyp19a1a negatively regulates IFN expression

**Fig 3:**
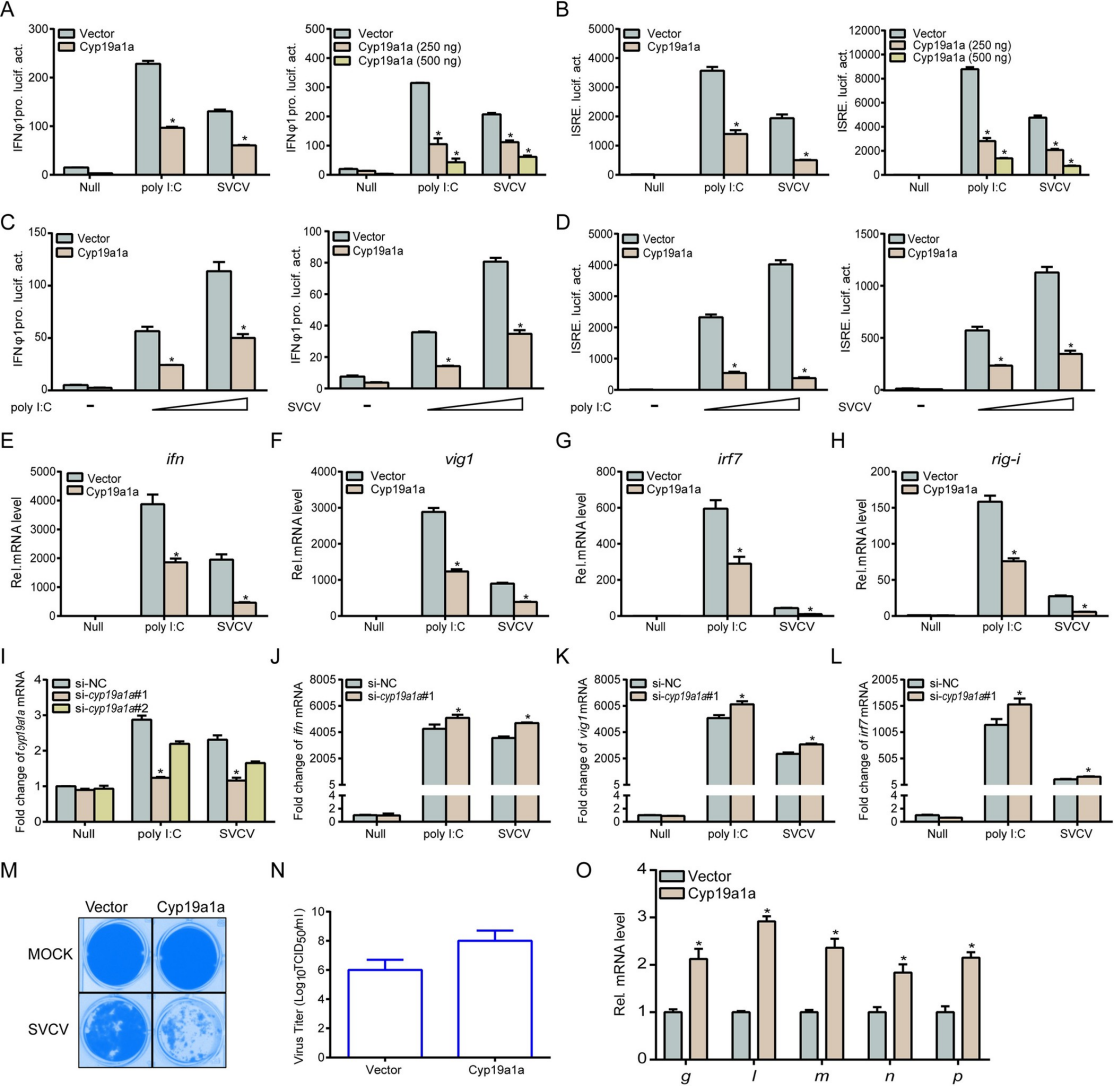
Overexpression of Cyp19a1a inhibits IFN induction and attenuates the cellular antiviral response. (A-D) Transfected with 250 ng IFNφ1pro (A and C) or ISRE-Luc (B and D) and 25 ng pRL-TK, plus Cyp19a1a-Flag (250 ng or 250/500 ng) or pCMV-Tag2C (control vector). Cells were transfected with poly I:C (0.5 μg or 0.5/1 μg), or treated with SVCV (MOI = 1 or 1/10). Luciferase activities were monitored. The promoter activity is presented as relative light units (RLU) normalized to *Renilla* luciferase activity. (E-H) Cyp19a1a suppresses the expression of *epc ifn* (E), *epc vig1* (F), *epc irf7* (G), and *epc rig-i* (H) induced by poly I:C or SVCV. Cells were transfected with 2 μg Cyp19a1a-Flag and transfected with poly I:C (2 μg) or treated with SVCV (MOI = 1) at 24 h post-transfection. Total RNAs were extracted for further qPCR assays. (I) Transfected with 100 nM si-*cyp19a1a*#1, si-*cyp19a1a*#2, or si-NC (negative control). At 24 h post-transfection, the cells were transfected with poly I:C (2 μg) or treated with SVCV (MOI = 1), total RNAs were extracted to examine the transcriptional levels of *cyp19a1a*. (J-L) EPC cells were transfected with 100 nM si-NC or si-*cyp19a1a*#1. At 24 h post-transfection, cells were untreated or transfected with poly I:C or treated with SVCV for 24 h before qPCR analysis was performed. The relative transcriptional levels were normalized to the transcription of *β-actin* and represented as fold induction relative to the transcriptional level in control cells, which was set to 1. (M and N) Transfected with 0.5 μg Cyp19a1a-Flag, at 24 h post-transfection, EPC cells were infected with SVCV (MOI = 0.001) for 48 h. Then, cells were fixed and stained (M). The viral titer of the culture supernatants from the cells infected with SVCV was measured according to the method of Reed and Muench (N). (O) Transfected with 2 μg of Cyp19a1a-Flag, at 24 h post-transfection, EPC cells were infected with SVCV (MOI = 1), total RNAs were extracted for further qPCR assays. The relative transcriptional levels were normalized to the transcriptional level of the *β-actin* gene and were represented as fold induction relative to the transcriptional level in control cells, which was set to 1. Data were expressed as mean ± SEM, n = 3. Asterisks indicate significant differences from control values (*, *p* < 0.05).

Since higher IFN activation is observed in *cyp19a1a*^-/-^ zebrafish, therefore, regulation of IFN activation by Cyp19a1a was verified next. Overexpression of Cyp19a1a significantly decreased poly I:C or SVCV mediated IFN and IFN-stimulated response element (ISRE) activation in a dose-dependent manner (Fig 3A and 3B). Stimulation with different stimulator dosages, also effectively induced the inhibition by Cyp19a1a (Fig 3C and 3D). At the mRNA level, Cyp19a1a suppressed transcription of *ifn* and several IFN-stimulated genes (ISGs) upon stimulation with poly I:C or SVCV (Fig 3E–3H). Specific *cyp19a1a* siRNA (si-*cyp19a1a*#1) was designed and used to more effectively knockdown the gene in following assays (Fig 3I). Stimulation of the *cyp19a1a* knockdown cells with poly I:C or SVCV, upregulated the transcription of *ifn* and ISGs compared to those in the control group (Fig 3J–3L). Furthermore, upon SVCV infection, Cyp19a1a evidently impaired the antiviral response capacity, with more cytopathogenic effect (CPE) observed in Cyp19a1a-overexpressed cells with dramatically increased SVCV titers (91-fold) compared to those of the control group (from 10^6.25^ to 10^8.21^ TCID_50_/ml at 48 h post-infection) (Fig 3M and 3N). At the molecular level, monitoring viral gene transcription revealed that Cyp19a1a significantly promoted viral gene replication (Fig 3O). These results suggested that Cyp19a1a decreases cellular IFN production in response to viral infection.

### Cyp19a1a interacts with MITA to inhibit MITA-mediated IFN activation

**Fig 4:**
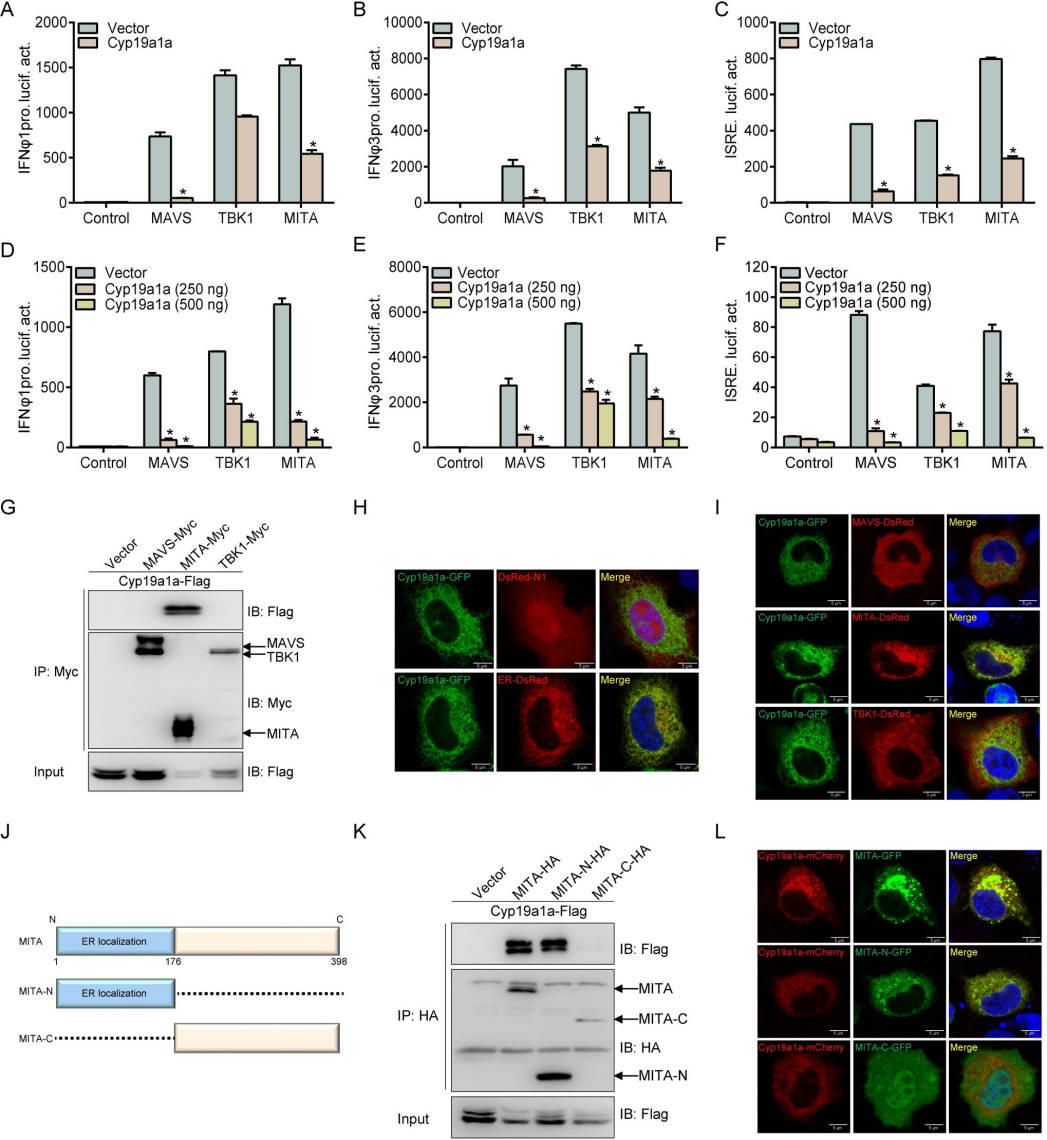
Cyp19a1a interacts with and blocks MITA-mediated IFN activation. (A-F) Co-transfected with MAVS-, TBK1-, MITA-expressing plasmid and empty vector or Cyp19a1a-Flag (250 ng or 250/500 ng), plus IFNφ1pro-Luc (A and D) or IFNφ3pro-Luc (B and E) or ISRE-Luc (C and F) at the ratio of 1:1:1, pRL-TK was used as a control. At 24 h post-transfection, cells were lysed for luciferase activity detection. Data were expressed as mean ± SEM, n = 3. Asterisks indicate significant differences from control (*, *p* < 0.05). (G) EPC cells were transfected with the indicated plasmids (5 μg each). After 24 h, cell lysates were immunoprecipitated (IP) with anti-Myc affinity gel. Then the immunoprecipitates and WCLs were analyzed by IB with the anti-Myc and anti-Flag Abs, respectively. (H and I) EPC cells were co-transfected with 1 μg Cyp19a1a-GFP plus 1 μg empty vector, ER-DsRed (H) and MAVS-DsRed/TBK1-DsRed/MITA-DsRed (I). After 24 h, the cells were fixed and subjected to confocal microscopy analysis. Green signals represent overexpressed Cyp19a1a, red signals represent overexpressed ER or RLRs. The yellow staining in the merged image indicates the colocalization of Cyp19a1a and ER or MITA (original magnification 63×; oil immersion objective). Scale bar, 10 μm. (J) Schematic representation of full-length MITA and two mutants (MITA-N containing the ER localization domain and MITA-C containing the C-terminal 222 amino acids). (K) EPC cells were co-transfected with the indicated plasmids (5 μg each). After 24 h, cell lysates were IP with anti-HA affinity gel. Then the immunoprecipitates and WCLs were analyzed by IB with the indicated Abs. (L) EPC cells were co-transfected with 1 μg Cyp19a1a-mCherry plus 1 μg MITA-GFP, MITA-N-GFP, or MITA-C-GFP. After 24 h, the cells were fixed and subjected to confocal microscopy analysis. Red signals represent overexpressed Cyp19a1a, green signals represent overexpressed MITA and its mutants. The yellow staining in the merged image indicates the colocalization of Cyp19a1a and MITA-N (original magnification 63×; oil immersion objective). Scale bar, 10 μm. All experiments were repeated for at least three times with similar results.

RLR signaling pathways have been reported to be crucial for IFN activation in fish; therefore, we investigated whether Cyp19a1a inhibits IFN expression through this signaling pathway. MAVS, TBK1, and MITA dramatically promoted IFN and ISRE promotor activities, which were dramatically inhibited by Cyp19a1a and display a dose-dependent manner (Fig 4A–4F). Subsequently, the interactions between Cyp19a1a and RLRs were analyzed at the protein level. Upon co-transfection with Cyp19a1a-Flag and Myc-tagged RLR molecules, including MAVS, TBK1, and MITA, the anti-Myc Ab-immunoprecipitated protein MITA was recognized by the anti-Flag Ab, indicating that the Cyp19a1a interacts with MITA (Fig 4G). Furthermore, the subcellular localizations of Cyp19a1a were monitored. Confocal microscopy analysis revealed that the green signals of Cyp19a1a overlapped with the red signals of the endoplasmic reticulum (ER) marker, suggesting that Cyp19a1a colocalizes at the ER (Fig 4H). Meanwhile, unlike the cytoplasmic localizations of MAVS and TBK1, the ER protein MITA was observed to colocalize with Cyp19a1a (Fig 4I). Next, the functional domain of MITA that interacts with Cyp19a1a was determined by generating two MITA mutants with truncations, MITA-N (lacking the C terminus) and MITA-C (lacking the N-terminal/ER localization region) (Fig 4J). Similar to wild-type MITA, MITA-N was able to bind with Cyp19a1a, whereas no interaction was observed with MITA-C (Fig 4K). Subcellular localization analysis confirmed that unlike MITA-C, MITA-N was localized at the ER similar to the wild type, and entirely overlapped with Cyp19a1a, indicating their colocalization (Fig 4L). Collectively, these results suggested that Cyp19a1a interacts with the N terminus of MITA to suppress MITA-mediated IFN activation.

### Cyp19a1a mediates autophagic degradation of MITA

**Fig 5:**
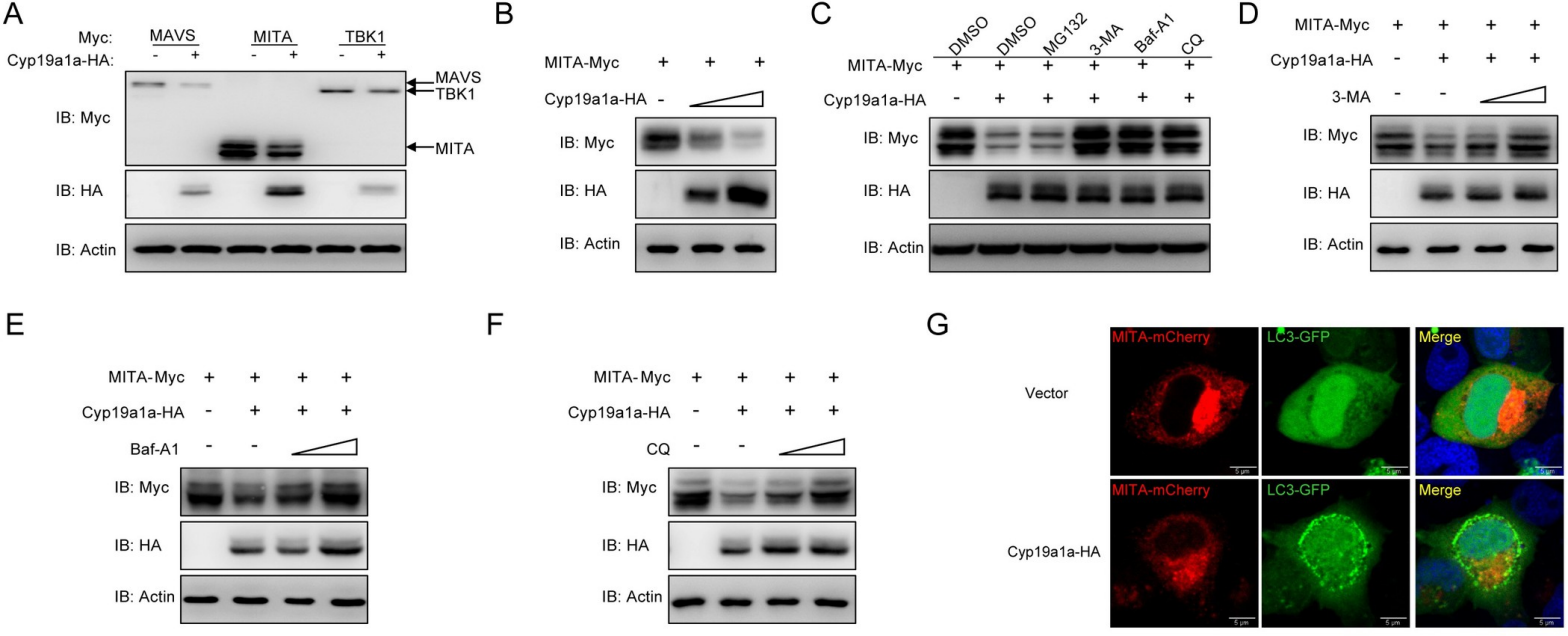
Cyp19a1a mediates degradation of MITA via the autophagy pathway. (A and B) EPC cells were co-transfected with 1 μg Cyp19a1a-HA and 1 μg empty vector, MAVS-Myc, MITA-Myc, or TBK1-Myc (A), or 1 μg MITA-Myc plus Cyp19a1a-HA (1 or 2 μg) or 2 μg empty vector (B) for 24 h. The cell lysates were subjected to IB with anti-Myc, anti-HA, and anti-β-actin Abs. (C) EPC cells were co-transfected the indicated plasmids. At 18 h post-transfection, the cells were treated with DMSO, MG132 (20 μM), 3-MA (2 mM), Baf-A1 (100 nM), or CQ (100 μM) for 6 h. The cell lysates were subjected to IB with the indicated Abs. (D-F) EPC cells were co-transfected the indicated plasmids. At 18 h post-transfection, the cells were treated with 3-MA (1 or 2 mM), Baf-A1 (50 or 100 nM), or CQ (50 or 100 μM) for 6 h. Then, the cells were harvested for IB with the indicated Abs. (G) EPC cells were co-transfected with 1 μg Cyp19a1a-HA or pCMV-HA plus 1 μg MITA-mCherry and 1 μg LC3-GFP. After 24 h, the cells were fixed and observed by confocal microscopy. Red signals represent overexpressed MITA, green signals represent overexpressed LC3 or GFP-LC3 positive autophagosome accumulation (original magnification 63×; oil immersion objective). Scale bar, 10 μm. All experiments were repeated for at least three times with similar results.

After elucidating the interaction between MITA and Cyp19a1a, the protein regulation pattern was investigated. In contrast to its minor effect on MAVS and TBK1 expression, Cyp19a1a dramatically reduced MITA stability in a dose-dependent manner (Fig 5A and 5B). In order to examine the exact regulation mechanism, several reagents including 3-Methyladenine (3-MA), bafilomycinA1 (Baf-A1), and chloroquine (CQ) were employed, which inhibit the ubiquitin (Ub)-proteasome, autophagosome, and lysosomal pathways during the late stage of autophagy, respectively. Cyp19a1a-mediated degradation of MITA was completely blocked by the autophagy-related inhibitors 3-MA, Baf-A1, and CQ, but not by MG132 (Fig 5C). Furthermore, MITA expressions were gradually rescued by increasing concentrations of 3-MA, Baf-A1, and CQ (Fig 5D–5F). Furthermore, upon lipidation, conjugated LC3 coats the autophagosome membrane; therefore, observing GFP-LC3 puncta formation was considered the autophagy indicator. At the cellular level, confocal microscopy analysis revealed that the LC3 puncta were notably accumulated in cells with overexpressed Cyp19a1a and MITA (Fig 5G). Taken together, these results suggested that Cyp19a1a degrades MITA through the autophagosome and lysosomal pathways.

### Simultaneous expression of Cyp19a1a and MITA is necessary for autophagosome formation

**Fig 6:**
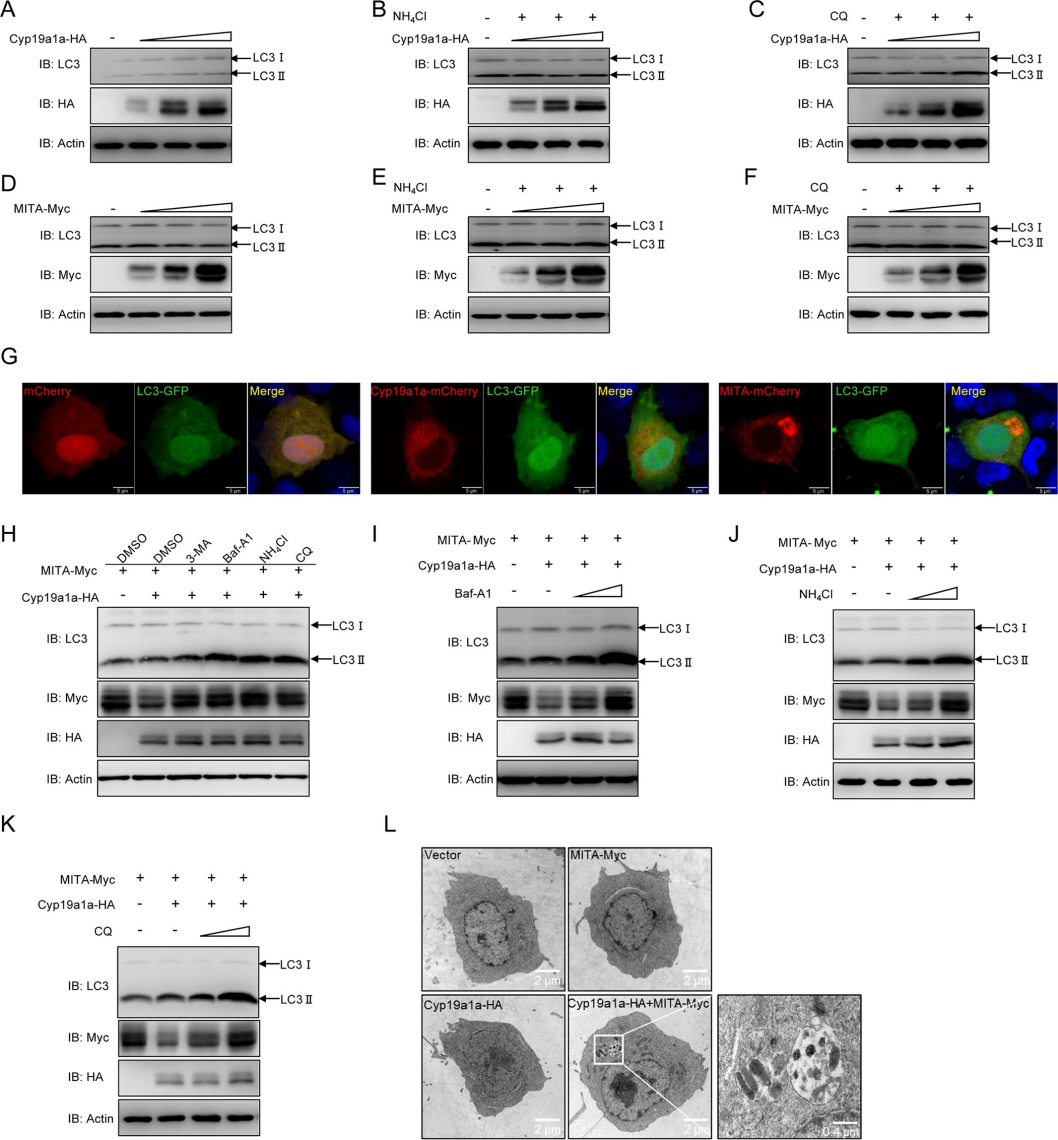
Co-transfection of Cyp19a1a and MITA triggers autophagy. (A-F) EPC cells were transfected with Cyp19a1a-HA (0.5, 1, or 2 μg) (A-C) or MITA-Myc (0.5, 1, or 2 μg) (D-F). At 18 h post-transfection, the cells were treated with DMSO, NH_4_Cl (20 mM), or CQ (100 μM) for 6 h. Then, the cells were harvested for IB with the indicated Abs. (G) EPC cells were co-transfected with 1 μg pCS2-mCherry, Cyp19a1a-mCherry, or MITA-mCherry plus 1 μg LC3-GFP. After 24 h, the cells were fixed and observed by confocal microscopy. Red signals represent overexpressed Cyp19a1a or MITA, green signals represent overexpressed LC3 (original magnification 63×; oil immersion objective). Scale bar, 10 μm. (H) EPC cells were co-transfected the indicated plasmids. At 18 h post-transfection, the cells were treated with DMSO, 3-MA (2 mM), Baf-A1 (100 nM), NH_4_Cl (20 mM), or CQ (100 μM) for 6 h. The cell lysates were subjected to IB with the anti-LC3, anti-Myc, anti-HA, and anti-β-actin Abs, respectively. (I-K) EPC cells were co-transfected the indicated plasmids. At 18 h post-transfection, the cells were treated with DMSO, Baf-A1 (50 or 100 nM), NH_4_Cl (10 or 20 mM), or CQ (50 or 100 μM) for 6 h. The cell lysates were subjected to IB with the indicated Abs. (L) EPC cells were transfected with indicated plasmids for 24 h. Then the cells were analyzed for autophagosome formation using a transmission electron microscope. Images on the right correspond to the squared zoomed images in each panel. Scale bar, 2 μm.

MITA is degraded by Cyp19a1a through the autophagic pathway; therefore, we examined whether Cyp19a1a causes cellular autophagy. Cellular lipidized LC3-II levels were determined in cells overexpressing Cyp19a1a. Intriguingly, LC3-II was unaffected by different expression levels of Cyp19a1a (Fig 6A). Next, to avoid the possibility that Cyp19a1a-induced host autophagic process is completed before samples are harvested, leading to undetectable changes of LC3-II, the cells were examined by monitoring the LC3-II level after treatment with NH_4_Cl and CQ. Consistent with the aforementioned results, the abundance of LC3-II did not change with NH_4_Cl and CQ treatment, suggesting that Cyp19a1a alone cannot induce host autophagy (Fig 6B and 6C). Similarly, overexpression of MITA alone did not affect LC3-II level under normal state or with reagent treatment (Fig 6D–6F). Meanwhile, cells overexpressing either Cyp19a1a or MITA alone, exhibited diffused distribution of LC3-GFP with only a few LC3 puncta observed (Fig 6G). Therefore, we speculate that both MITA and Cyp19a1a are essential for autophagy. As expected, co-overexpression of Cyp19a1a and MITA dose-dependently increased LC3-II levels in Baf-A1-, NH_4_Cl-, and CQ-treated groups (Fig 6H–6K). Meanwhile, autophagosome-like vesicles with cytosolic contents were observed in the presence of Cyp19a1a and MITA; whereas they were absent in cells overexpressing either Cyp19a1a or MITA alone (Fig 6L). These data suggested that simultaneous expression of Cyp19a1a and MITA is necessary to initiate host autophagy that degrades MITA.

### ATG14 is essential for Cyp19a1a-mediated autophagic degradation of MITA

**Fig 7:**
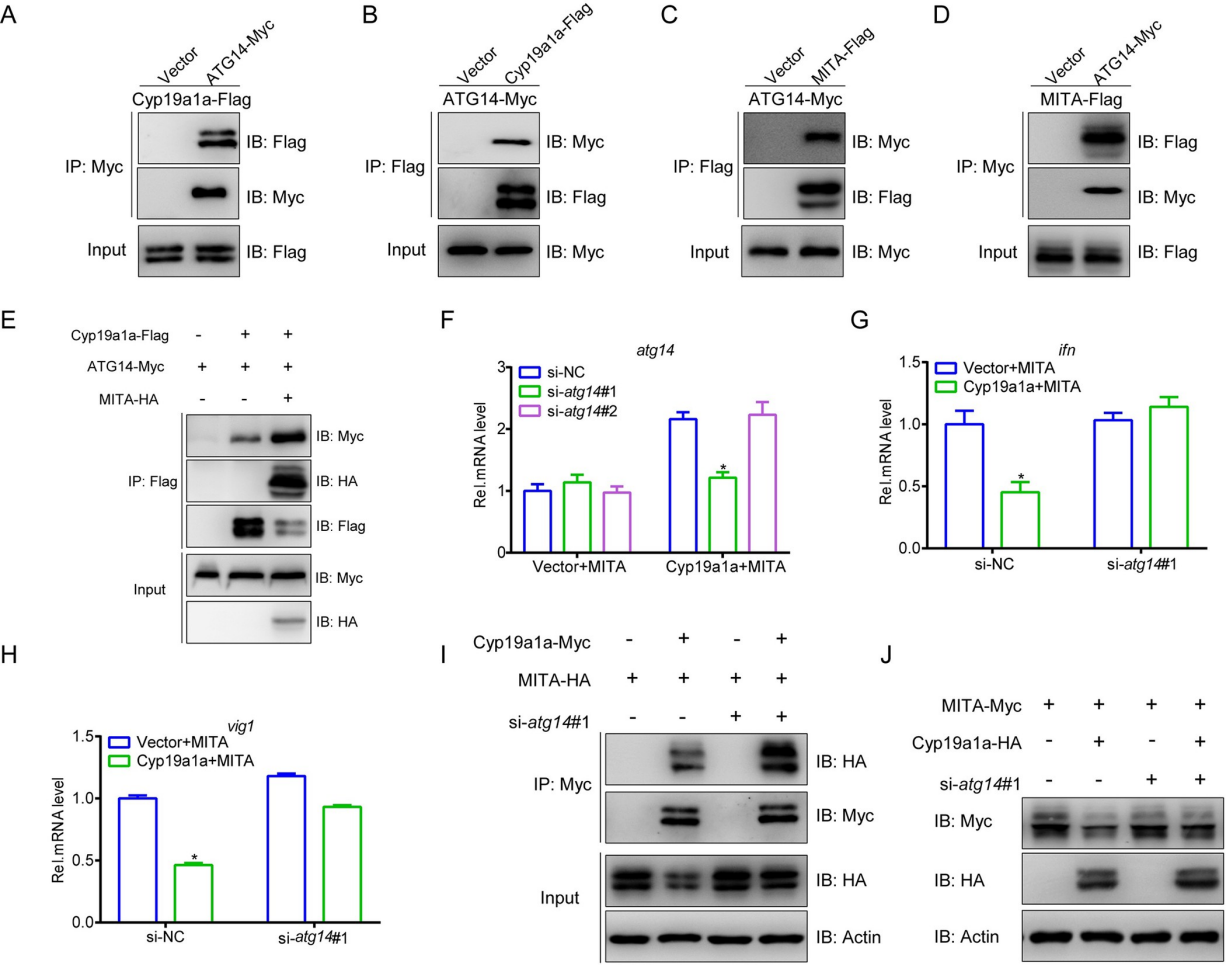
ATG14 is essential for Cyp19a1a mediated MITA autophagic degradation. (A-D) EPC cells were transfected with the indicated plasmids (5 μg each). After 24 h, cell lysates were IP with anti-Myc or anti-Flag affinity gel. Then the immunoprecipitates and WCLs were analyzed by IB with the anti-Myc and anti-Flag Abs, respectively. (E) EPC cells were transfected with the indicated plasmids (4 μg each). After 24 h, cell lysates were IP with anti-Flag affinity gel. Then the immunoprecipitates and WCLs were analyzed by IB with the anti-Myc, anti-HA, and anti-Flag Abs, respectively. (F) EPC cells were transfected with 100 nM si-*atg14*#1, si-*atg14*#2, or si-NC (negative control). At 6 h post-transfection, the cells were transfected with 1.5 μg MITA-Myc plus 1.5 μg Cyp19a1a-Flag or empty vector. At 24 h post-transfection, total RNAs were extracted to examine the transcriptional levels of ATG14. (G and H) EPC cells were transfected with 100 nM si-NC or si-*atg14*#1. At 24 h post-transfection, cells were co-transfected with 1.5 μg MITA-Myc and 1.5 μg Cyp19a1a-Flag or empty vector for 24 h before qPCR analysis was performed. The relative transcriptional levels were normalized to the transcription of *β-actin* and represented as fold induction relative to the transcriptional level in control cells, which was set to 1. (I) EPC cells were transfected with 100 nM si-NC or si-*atg14*#1. After 24 h, the cells were transfected by the indicated plasmids (5 μg each) for 24 h. Cell lysates were IP with anti-Myc affinity gel. Then the immunoprecipitates and WCLs were analyzed by IB with the indicated Abs. (J) EPC cells were transfected with 100 nM si-NC or si-*atg14*#1. At 24 h post-transfection, cells were co-transfected with 1.5 μg MITA-Myc plus 1.5 μg Cyp19a1a-HA or empty vector for 24 h. The cell lysates were subjected to IB with anti-Myc, anti-HA, and anti-β-actin Abs.

Large numbers of autophagy-related (ATG) proteins have been shown to drive autophagosome formation to degrade target proteins [27]. In order to delineate the mechanisms of Cyp19a1a-mediated autophagic degradation of MITA, we cloned and screened several ATG members; ATG14 was selected for further experimentation. First, the protein interaction was identified between ATG14 and Cyp19a1a or MITA. The anti-Myc Ab-immunoprecipitated protein ATG14 was recognized by the anti-Flag Ab, demonstrating that Cyp19a1a interacts with ATG14 (Fig 7A). This was confirmed by the results of the reverse immunoprecipitation (IP) assay (Fig 7B). Meanwhile, the interactions of MITA with ATG14 were confirmed by Co-IP (Fig 7C and 7D). Interestingly, the recruitment of ATG14 by Cyp19a1a was significantly enhanced upon the participation of MITA, suggesting that simultaneous presence of Cyp19a1a and MITA promotes their interaction with ATG14 (Fig 7E). This phenomenon is also indirectly demonstrated by the increased LC3-II level and presence of autophagosome-like vesicles when both Cyp19a1a and MITA were present. Finally, an siRNA against *atg14* was designed for subsequent assays, the inhibition of Cyp19a1a on MITA-mediated IFN and ISGs induction was rescued by knockdown *atg14* (Fig 7F–7H). In addition, the interaction of MITA and Cyp19a1a was also increased by knockdown *atg14* which was confirmed by Co-IP, meanwhile, at the protein level, the degradation of MITA induced by Cyp19a1a was notably impaired when *atg14* was knocked down (Fig 7I and 7J). These results indicated that ATG14 is an essential factor for Cyp19a1a-mediated autophagic degradation of MITA.

### Cyp19a1a dampens the MITA-mediated cellular antiviral response

**Fig 8:**
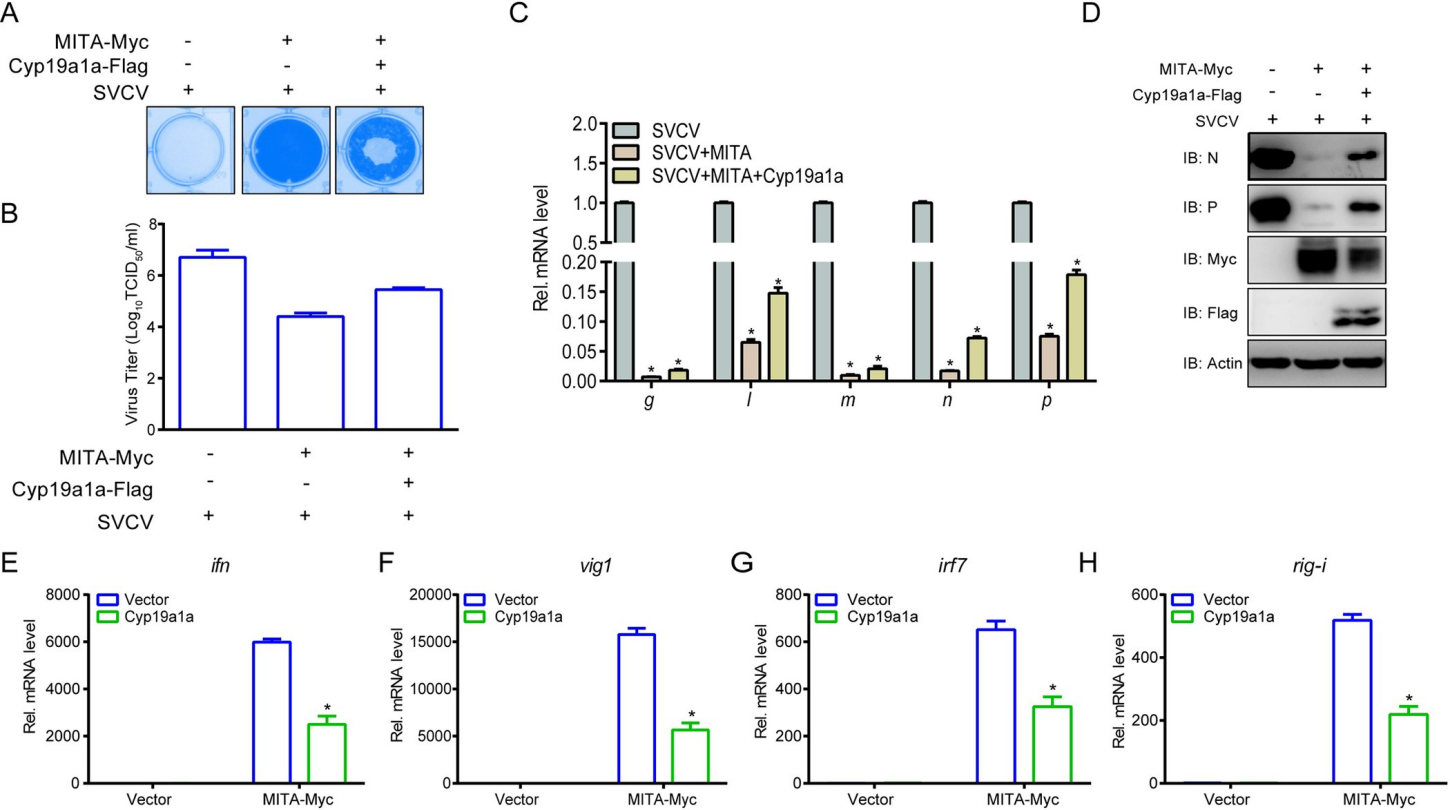
Cyp19a1a blocks MITA-mediated cellular antiviral response. (A and B) EPC cells were transfected with 0.25 μg MITA-Myc and 0.25 μg Cyp19a1a-Flag or empty vector. At 24 h post-transfection, cells were infected with SVCV (MOI = 0.001) for 48 h. Then, cells were fixed with 4% PFA and stained with 1% crystal violet (A). Culture supernatants from the cells infected with SVCV were collected, and the viral titer was measured according to the method of Reed and Muench (B). (C) EPC cells were transfected with 1.5 μg Cyp19a1a-Flag or empty vector together with 1.5 μg MITA-Myc or empty vector. At 24 h post-transfection, cells were infected with SVCV (MOI = 1). After 24 h-infection, total RNAs were extracted to examine the mRNA levels of cellular *g*, *l*, *m*, *n*, and *p*. (D) The same samples were prepared similarly as described above for panel C. The lysates were detected by IB with anti-N, anti-P, anti-Myc, anti-Flag, and anti-β-actin Abs, respectively. (E-H) Overexpression of Cyp19a1a suppresses the expression of *epc ifn (E)*, *epc vig1* (F), *epc irf7* (G), and *epc rig-i* (H) induced by MITA. EPC cells were transfected with 1.5 μg Cyp19a1a-Flag or empty vector together with 1.5 μg MITA-Myc or empty vector. At 24 h after transfection, total RNAs were extracted for further qPCR assays. The relative transcriptional levels were normalized to the transcriptional level of the *β-actin* gene and were represented as fold induction relative to the transcriptional level in the control cells, which was set to 1. Data were expressed as mean ± SEM, n = 3. Asterisks indicate significant differences from control values (*, *p* < 0.05).

Cyp19a1a targets MITA for autophagic degradation, which is known to be a strong antiviral factor; therefore, we examined whether Cyp19a1a influences MITA-mediated cellular antiviral response capacity. Upon viral infection, MITA protected the cells and reduced the viral titer 188-fold (from 10^6.7^ to 10^4.425^ TCID_50_/ml at 48 h post-infection), whereas Cyp19a1a interfered with the antiviral function of MITA in terms of both CPE and viral titers (from 10^4.425^ to 10^5.45^ TCID_50_/ml at 48 h post-infection) (Fig 8A and 8B). Subsequently, at both gene and protein level, the effect of MITA on virus inhibition was weakened by Cyp19a1a (Fig 8C and 8D). For host antiviral IFN response, MITA-induced upregulation of ISGs was also reversed by Cyp19a1a (Fig 8E–8H). These results demonstrated that the cellular antiviral response capacity induced by MITA is attenuated by Cyp19a1a.

## Discussion

It has been well documented that sex drives dimorphic immune responses to viral infections in various species, including mammals and birds [4]. Epidemiological studies have shown that females exhibit stronger innate and adaptive immune responses than males during viral infection [28]. Unlike in mammals, gonadal differentiation in fish displays greater diversity and plasticity [29]. Therefore, there is not much research published on the differences between male and female fish in response to viral infection. In recent years, numerous studies have shown that *cyp19a1a* is crucially involved in regulating female development and ovarian differentiation in teleosts. In this study, we found that male zebrafish display stronger antiviral responses than females. Further mechanistic studies revealed that this phenomenon was because of high Cyp19a1a expression in female fish, which in-turn inhibits IFN expression through autophagic-lysosomal degradation of MITA.

Studies in humans and animal models indicate that females exhibit stronger innate, cellular, and humoral immune responses; however, they are more susceptibility to autoimmune diseases than males. Unexpectedly, we found that male fish are more resistant to viral infections than females. Research in marine medaka has revealed that females are more susceptibility to bacteria *Edwardsiella tarda* infection-induced mortality than males [30]. However, these results are totally opposite to what has been reported in mammals. Commonly, fish sex breeding is to generate monosex populations (all-female or all-male) based on growth rate and body size [31,32]. Our findings provide another important trait, resistance to viral infections, for sex control breeding in aquaculture.

In our study, female fish with high endogenous Cyp19a1a levels were vulnerable to SVCV infection. Conversely, males with low levels of Cyp19a1a were more resistant to SVCV infection than females. In the marine medaka model, the sexual dimorphism in disease survival is particularly obvious because the immune competence decreases in a sex-dependent manner during spawning [33]. Our data demonstrated that endogenous Cyp19a1a is among the crucial factors responsible for sex-specific immune responses, further emphasizing the need to assess Cyp19a1a as a negative regulator of IFN with more pronounced effects in females.

MITA has been established as a signaling hub that exerts its various functions in a molecular context-dependent manner [34]. On the one hand, MITA functions as a direct sensor for cyclic dinucleotides and as an adaptor protein to recruit downstream signaling components, thereby facilitating signal transduction. On the other hand, MITA is involved in regulating mainstream cellular programs, such as differentiation, proliferation, and programmed cell death [21]. Consequently, MITA is a preferred target to reduce excessive immune response. It has been proved that fish MITA predominantly localizes at the ER and positively regulates IFN and ISG induction [26]. Interestingly, we found that Cyp19a1a also localized to the ER and interacted with MITA, whether the ER localization of MITA is affected by Cyp19a1a still needs to be investigated.

The autophagosome-lysosome pathway and the ubiquitin-proteasome system are two major degradation mechanisms that remove abnormal and unused proteins to maintain intracellular protein homeostasis [35]. Only the ubiquitin-proteasome pathway has been reported to mediate MITA degradation in previous studies. For instance, tripartite motif containing 29 (TRIM29) has been shown to target MITA for K48-linked ubiquitination and degradation [36]. Our results support the involvement of the autophagosome-lysosome system in MITA degradation. In this work, we have demonstrated that Cyp19a1a recruits more ATG14 to combine with MITA, suggesting that Cyp19a1a may sequester MITA to autophagosomes through ATG14, leading to suppression of IFN expression. Further investigations are required to understand how Cyp19a1a and ATG14 cooperate to degrade MITA.

Overall, this study provides substantial insights into sexual dimorphism in immune responses to viral infections. Aromatase Cyp19a1a as a negative regulator of IFN controls its production in a sex-dependent manner. This has been reported for the first time in fish. The observed sex-differences in disease resistance should to be integrated into the future assessment of immune endpoints in sexually mature fish.

## Materials and methods

### Ethics statement

*In vivo* experiments of fish described in the present study were conducted at the Institute of Hydrobiology (IHB), Chinese Academy of Sciences (CAS) according to European Union guidelines for handling of laboratory animals (2010/63/EU). The whole study was in compliance with all ethical regulations and was approved by the Committee on the Ethics of Animal Experiments of the IHB, CAS (No. 2019–040).

### Fish

AB line wild type zebrafish (*Danio rerio*) and cyp19a1a mutant zebrafish (CZ307: *cyp19a1a*^*ihb158/+*^) were provided by the China Zebrafish Resource Center (CZRC). The breeding and maintenance of zebrafish were performed according to standard methods [37]. The mature zebrafish individuals used for experiments were about 2.5 months post hatching (0.4 ± 0.1g). Hexaploid gibel carp (*Carassius gibelio*) and red common carp (*Cyprinus carpio*) were provided by the National Aquatic Biological Resource Center (NABRC). A maternal gibel carp from strain A^+^ was mated with a paternal red common carp to initiate gynogenesis, in which the unreduced eggs were stimulated by the sperm to activate embryogenesis inheriting only maternal genetic information [38]. One part of the gynogenetic larvae were raised at normal temperature (20 ± 1°C) to produce all-female offspring, while the other of the same family were raised at high temperature (30 ± 1°C) to produce all-male offspring as the sex of gibel carp could be affected by the ambient temperature during larval development [39]. The gibel carp individuals used for experiments were about 2.5 months post hatching (3 ± 1 g). The breeding and maintenance of gibel carp were performed as previously described [40]. All fish were maintained according to institutional and national ethical and animal welfare guidelines in the laboratory for at least two weeks prior to the experiments for acclimatization and evaluation of overall fish health. Only the healthy fish, as determined by the general appearance and the level of activity, were used for studies. To avoid the gene expression based on circadian rhythm, all infection experiment of both male and female fish was completed within 30 min [41].

### Cells and viruses

Zebrafish liver (ZFL) cells were obtained from a pool of 10 adult zebrafish livers, whose sex were not recorded (American Type Culture Collection, ATCC, www.atcc.com). ZFL cells were cultured at 28°C in 5% CO_2_ in Ham’s F12 nutrient mixture medium (Invitrogen) supplemented with 10% fetal bovine serum (FBS, Invitrogen). Epithelioma papulosum cyprini (EPC) cells were derived from fathead minnow (*Pimephales promelas*) without sex record [42], which is belongs to *Cyprinidae* and is also phylogenetically closely related to zebrafish. EPC cells were obtained from China Center for Type Culture Collection (CCTCC) and were maintained at 28°C in 5% CO_2_ in medium 199 (M199) (Invitrogen) supplemented with 10% FBS. SVCV, a negative ssRNA virus, was propagated in EPC cells until a CPE was observed, and then cell culture fluid containing SVCV was harvested and centrifuged at 4 × 10^3^ *g* for 20 min to remove the cell debris, and the supernatant was stored at -80°C until used. *Ca*HV was provided by Prof. Q. Y. Zhang (Institute of Hydrobiology, Chinese Academy of Sciences) and propagated by intraperitoneal injection into healthy gibel carp. The isolation method of *Ca*HV was used as previously described [43].

### Plasmid construction and reagents

The sequence of Cyp19a1a (ZDB-GENE-990415-43) was obtained from the ZFIN website (https://zfin.org/). Using the cDNA of the tissues from zebrafish as template, the open reading frame (ORF) of Cyp19a1a were amplified by polymerase chain reaction (PCR) and cloned into pCMV-Myc or pCMV-Tag2C vectors (Clontech), respectively. The ORFs of zebrafish MAVS (ZDB-GENE-070112-1402), TBK1 (ZDB-GENE-060512-359), MITA (ZDB-GENE-120921-1), and the truncated mutants of MITA, and ATG14 (ZDB-GENE-050913-155) were also subcloned into pCMV-Myc, pCMV-HA, and pCMV-Tag2C vectors (Clontech), respectively. For subcellular localization, the ORF of zebrafish Cyp19a1a was inserted into pEGFP-N3 or pCS2-mCherry vectors (Clontech). The ORFs of MAVS, TBK1, MITA, MITA mutants, and LC3 (ZDB-GENE-030131-1145) were also inserted into pEGFP-N3, pCS2-mCherry, or pDsRed-N1 vectors (Clontech). The pDsRed-ER plasmid was purchased from Clontech. The plasmids containing zebrafish IFNφ1pro-Luc, IFNφ3pro-Luc, and ISRE-Luc in the pGL3-Basic luciferase reporter vector (Promega) were constructed as described previously [44,45]. The *Renilla* luciferase internal control vector (pRL-TK) was purchased from Promega. All constructs were confirmed by DNA sequencing. The primers including the restriction enzyme cutting sites used for plasmid construction are listed in S1 Table. Poly I:C was purchased from Sigma-Aldrich used at a final concentration of 1 μg/μl. MG132 (Cat. No. M7449), 3-MA (Cat. No. M9281), NH_4_Cl (Cat. No. A9464), CQ (Cat. No. C6628) were obtained from Sigma-Aldrich. Baf-A1 (Cat. No. S1413) was obtained from Selleck.

### Luciferase activity assay

EPC cells were seeded in 24-well plates overnight and co-transfected with the indicated luciferase reporter plasmids and expression vectors. The empty vector was used to ensure equivalent amounts of total DNA in each well. Stimulation of poly I:C or SVCV infection was performed at 24 h post-transfection. At 24 h post-stimulation, the cells were washed with phosphate-buffered saline (PBS) and lysed for measuring luciferase activity by the Dual-Luciferase Reporter Assay System (Promega) according to the manufacturer’s instructions. Firefly luciferase activity was normalized on the basis of *Renilla* luciferase activity.

### Transient transfection and virus infection

Transient transfections were performed in EPC cells seeded in 6-well or 24-well plates by using FishTrans (MeiSenTe Biotechnology) according to the manufacturer’s protocol. For the antiviral assay, EPC cells seeded in 24-well plates were transfected with 0.5 μg Cyp19a1a-Myc or the empty vector. At 24 h post-transfection, cells were infected with SVCV (MOI = 0.001). After 48 h or 72 h, supernatant aliquots were harvested for detection of virus titers, the cell monolayers were fixed by 4% paraformaldehyde (PFA) and stained with 1% crystal violet for visualizing CPE. For virus titration, 200 μl of culture medium were collected at 48 h post-infection and used for detection of virus titers according to the method of Reed and Muench. The supernatants were subjected to 3-fold or 10-fold serial dilutions and then added (100 μl) onto a monolayer of EPC cells cultured in a 96-well plate. After 48 or 72 h, the medium was removed and the cells were washed with PBS, fixed by 4% PFA and stained with 1% crystal violet. The virus titer was expressed as 50% tissue culture infective dose (TCID_50_/ml). For viral infection, fish were anesthetized with tricaine methanesulfonate (MS-222) and i.p. injected with 5/10 μl of M199 containing SVCV (5 × 10^8^ TCID_50_/ml). As the control group, fish were treated similarly and injected i.p. with 5/10 μl of M199 collected from non-infected cells. Then the fish were migrated into the aquarium containing new aquatic water.

### RNA extraction, reverse transcription, and quantitative real-time PCR (qPCR)

For fish tissue samples, at 24 h or 48 h post-injection, all fish were anaesthetized with MS-222, dissected, and then gonads, spleens, and trunk kidneys were harvested and immediately frozen in liquid nitrogen and stored at −80°C for further qPCR assays. Total RNAs were extracted by the TRIzol reagent (Invitrogen). Genomic DNA was thoroughly digested by RNase free DNase (Promega). First-strand cDNA was synthesized by using a GoScript reverse transcription system (Promega) according to the manufacturer’s instructions. qPCR was performed with Fast SYBR green PCR master mix (Bio-Rad) on the CFX96 real-time system (Bio-Rad). PCR conditions were as follows: 95°C for 5 min and then 40 cycles of 95°C for 20 s, 60°C for 20 s, and 72°C for 20 s. All primers used for qPCRs are shown in S1 Table, and *β-actin* gene was used as an internal control. The relative fold changes were calculated by comparison to the corresponding controls using the 2^-ΔΔCt^ method. Three independent experiments were conducted for statistical analysis.

### RNA interference (RNAi)

EPC cells were seeded in 6-well plates overnight and transfected with 100 nM small interfering RNAs (siRNA) of *cyp19a1a* or *atg14* and the negative control (si-Nc) by using FishTrans (MeiSenTe Biotechnology). siRNA of *cyp19a1a* or *atg14* and si-Nc were obtained from GenePharma (Shanghai, China). The following sequences were targeted for EPC Cyp19a1a (OK019722): si-*cyp19a1a*#1: GCAGUGUAUUGGGAUGCAUTT; si-*cyp19a1a*#2: GCAGACAGUAUUAAUCAAATT. The following sequences were targeted for EPC ATG14 (MZ969009): si-*atg14*#1: GGAGAUUGCUCAGCCUAUUTT; si-*atg14*#2: GGUAGUGACGGAUGAAGAATT.

### Co-IP assay

For Co-IP experiments, EPC cells seeded in 10 cm^2^ dishes overnight were transfected with a total of 10 μg of the indicated plasmids. At 24 h post-transfection, the medium was removed carefully, and the cell monolayer was washed twice with 10 ml ice-cold PBS. Then the cells were lysed in 1 ml of radioimmunoprecipitation (RIPA) lysis buffer [1% NP-40, 50 mM Tris-HCl, pH 7.5, 150 mM NaCl, 1 mM EDTA, 1 mM NaF, 1 mM sodium orthovanadate (Na_3_VO_4_), 1 mM phenylmethylsulfonyl fluoride (PMSF), 0.25% sodium deoxycholate] containing protease inhibitor cocktail (Sigma-Aldrich) at 4°C for 1 h on a rocker platform. The cellular debris was removed by centrifugation at 12,000 × *g* for 15 min at 4°C. The supernatant was transferred to a fresh tube and incubated with 30 μl anti-Flag/HA/Myc affinity gel (Sigma-Aldrich) overnight at 4°C with constant rotating incubation. These samples were further analyzed by immunoblotting (IB). Immunoprecipitated proteins were collected by centrifugation at 5000 × *g* for 1 min at 4°C, washed three times with lysis buffer and resuspended in 50 μl 2 × SDS sample buffer. The immunoprecipitates and whole cell lysates (WCLs) were analyzed by IB with the indicated Abs.

### Immunoblot analysis

Immunoprecipitates or WCLs were separated by 10% SDS-PAGE and transferred to polyvinylidene difluoride (PVDF) membrane (Bio-Rad). The membranes were blocked for 1 h at room temperature in TBST buffer (25 mM Tris-HCl, 150 mM NaCl, 0.1% Tween 20, pH 7.5) containing 5% skim milk, and then probed with the indicated primary Abs at an appropriate dilution overnight at 4°C. After being washed three times with TBST, the membranes were incubated with secondary Abs for 1 h at room temperature. After three additional washes with TBST, the membranes were stained with the Immobilon Western chemiluminescent horseradish peroxidase (HRP) substrate (Millipore) and detected by using an ImageQuant LAS 4000 system (GE Healthcare). Abs were diluted as follows: anti-β-actin (ABclonal, AC026) at 1:3,000, anti-Flag (Sigma-Aldrich, F1804) at 1:3,000, anti-HA (Covance, MMS-101R) at 1:3000, anti-Myc (Santa Cruz Biotechnology, sc-40) at 1:3,000, anti-LC3 (Abcam, ab48394) at 1:1000, and HRP-conjugated anti-mouse/rabbit IgG (Thermo Scientific, 31430/31460) at 1:5,000. Results are representative of three independent experiments.

### Transmission electron microscopy (TEM)

EPC cells were seeded in 6-well plates and transfected with indicated plasmids for 24 h. For pretreatment, cells were washed three times with PBS, trypsinized, and transferred to 1.5 ml centrifuge tube. Cell precipitation was collected by centrifugation at 2000 × *g* for 5 min. The cell pellets were resuspended with 2.5% glutaraldehyde in 0.075 mol/L phosphate buffer (pH 7.4) for 4 h at 4°C for prefixation. Then the cells were washed three times with solution containing 0.075 mol/L phosphate and 0.19 mol/L sucrose for 15 min each and post-fixed in 1% osmium tetroxide (OsO_4_) in 0.24 mol/L phosphate buffer (pH 7.4) for 2 h. After being washed three times for 15 min each in 0.075 mol/L phosphate buffer and 0.19 mol/L sucrose buffer at 4°C, the cells were dehydrated with a graded series of ethanol and acetone, and then gradually infiltrated with epoxy resin. Samples were sequentially polymerized at 37°C overnight and then 60°C for 48 h. Ultrathin sections (74 nm) were cut using microtome (UC7; Leica) and mounted on copper slot grids. Sections were doubly stained with 3% uranyl acetate-lead citrate for 10 min and observed under transmission electron microscope (HT7700; Hitachi).

### Fluorescent microscopy

EPC cells were plated onto coverslips in 6-well plates and transfected with the plasmids indicated for 24 h. Then the cells were washed twice with PBS and fixed with 4% PFA for 1 h. After being washed three times with PBS, the cells were stained with 1 μg/ml 4′, 6-diamidino-2-phenylindole (DAPI; Beyotime) for 15 min in the dark at room temperature. Finally, the coverslips were washed and observed with a confocal microscope under a 63× oil immersion objective (SP8; Leica).

### Histopathology

Gonad, spleen, and heart tissues from three individuals of control or virus-infected fish at 3 days post infection (dpi) were dissected, and fixed in 10% phosphate-buffered formalin overnight. Then the samples were dehydrated in ascending grades of alcohol and embedded into paraffin. Sections at 5 μm thickness were taken and stained with hematoxylin and eosin (H&E). Histological changes were examined by optical microscopy at ×40 magnification and were analyzed by the Aperio ImageScope software.

### Statistics analysis

For fish survival analysis, Kaplan-Meier survival curves were generated and analyzed by Log-rank test. For the bar graph, one representative experiment of at least three independent experiments is shown, and each was done in triplicate. For the dot plot graph, each dot point represents one independent biological replicate. Unpaired Student’s t test was used for statistical analysis. Data are expressed as mean ± standard error of the mean (SEM). A *p* value < 0.05 was considered statistically significant.

## Supporting information

S1 Table Primers used in this study. (PDF)

S1 Fig Analysis of correlation between sex-related genes and IFN expression. (A) Transfected with 250 ng IFNφ1pro and 25 ng pRL-TK, plus 250 ng Cyp19a1a-Myc/Amh-Myc/Dmrt1-Myc/Foxl2a-Myc/Foxl2b-Myc/Sox9a-Myc/Sox9b-Myc or pCMV-Myc (control vector). After 24 h, cells were transfected with poly I:C (1 μg). Luciferase activities were monitored. The promoter activity is presented as relative light units (RLU) normalized to Renilla luciferase activity. (B-H) EPC cells were transfected with the indicated plasmids (5 μg each). After 24 h, cell lysates were IP with anti-Flag affinity gel. Then the immunoprecipitates and WCLs were analyzed by IB with the anti-Myc or anti-HA, and anti-Flag Abs, respectively.
(TIF)
