# Supplementary material for: Fish female-biased gene cyp19a1a leads to female antiviral response attenuation between sexes by autophagic degradation of MITA
Source: PLoS Pathog. 2022 Jun 21;18(6):e1010626. doi: 10.1371/journal.ppat.1010626 (PMC9249237; doi:10.1371/journal.ppat.1010626)
Supplement: S1 Table — (PDF) [file ppat.1010626.s001.pdf]

Table S1. Primers used in this study.

Name	Sequence (5'→3')	Purpose
pCMV-HA/Myc-Cyp19a1a-F	CGGGGTACCATGGCAGGTGATCTGCTCCAGCCCTGTGG	Eukaryotic expression
pCMV-HA/Myc-Cyp19a1a-R	AAGGAAAAAAGCGGCCGCTCAGAGAGTGTTTCTGAGGATAAGCTGC	
pCMV-Tag2c-cyp19a1a-F	GATATCAAATGGCAGGTGATCTGCTCCAGCCCTGTGG	
pCMV-Tag2c-Cyp19a1a-R	ACGCGTCGACTCAGAGAGTGTTTCTGAGGATAAGCTGC	
mCherry-Cyp19a1a-F	CGCGGATCCGCCACCATGGCAGGTGATCTGCTCC	
mCherry-Cyp19a1a-R	CCCATCGATGAGAGTGTTTCTGAGGATAAG	
EGFP-Cyp19a1a-F	CTAGCTAGCATGGCAGGTGATCTGCTCCAGCCCTGTGG	
EGFP-Cyp19a1a-R	CGGGGTACCGAGAGTGTTTCTGAGGATAAGCTGC	
pCMV-Myc/Tag2C- ATG14-F	CGGAATTCCG ATGGCTTGTCCCTCAACAGGC	
pCMV-Myc/Tag2C-ATG14-R	CCCTCGAGTCAGCGCTGGCCTGTGTAGGC	
DsRed-MAVS-F	CGCGGATCCGCCACCATGTCACTGACACGTGAGC	
DsRed-MAVS-R	CCCATCGATATGATTGAGCTTCCAG	
DsRed-TBK1-F	CGCGGATCCGCCACCATGCAGAGTACGGCCAAT	
DsRed-TBK1-R	CCGGAATTCCGCATCCGCTCCACTG	
DsRed-MITA-F	CGCGGATCCGCCACCATGTCTGTGATGGGAG	
DsRed-MITA-R	CCGGAATTCCGGTTTTGTTTCATTGC	
pCMV-Myc/Tag2C-MAVS-F	CCGCTCGAGCGATGTCACTGACACGTGAGC	
pCMV-Myc/Tag2C-MAVS-R	ATTTGCGGCCGCTTAATGATTGAGCTTCCAG	
pCMV-Myc/Tag2C-TBK1-F	CGCGGATCCCGATGCAGAGTACGGCCAAT	
pCMV-Myc/Tag2C-TBK1-R	AACTCGAGTCACATCCGCTCCACTG	
pCMV-Myc/Tag2C-MITA-F	CCGGAATTCCGATGTCTGTGATGGGAGAA	

pCMV-Myc/Tag2C-MITA-R	AACTCGAGTTAGTTTTGTTTCATTGC	
pEGFP-N3-MITA-F	CTAGCTAGCATGTCTGTGATGGGAG	
pEGFP-N3-MITA-R	CGCGGATCCGTTTTGTTTCATTGC	
pEGFP-N3-MITA-C-F	CTAGCTAGCATGGTGGCTCATGGTCTGG	
pEGFP-N3-MITA-N-R	CGCGGATCCGTTCATCTTTTTTGCTTC	
pCMV-HA-MITA-N-R	CCGCTCGAGTTAGTTCATCTTTTTTGCTTC	
pCMV-HA-MITA-C-F	CCGGAATTCCGATGGTGGCTCATGGTCTGG	
Dr ifnφ1-F	GAATGGCTTGGCCGATACAGGATA	
Dr ifnφ1-R	TCCTCCACCTTTGACTTGTCCATC	
Dr cyp19a1a-F	CTAGGAGTCCCGCTCAATGAGCAC	qPCR
Dr cyp19a1a-R	GGCATCCTGCAACTCCTGAGCATC	
Dr β-actin-F	TCCAGCCTTCCTTCCTGGGTATG	
Dr β-actin-R	TCAGGGGGAGCAATGATCTTGA	
epc ifn-F	ATGAAAACTCAAATGTGGACGTA	
epc ifn-R	GATAGTTTCCACCCTTTCCTTAA	
epc vig1-F	AGCGAGGCTTACGACTTCTG	
epc vig1-R	GCACCAACTCTCCCAGAAAA	
epc irf7-F	GGAGGACCAACACAAAGTCTATC	
epc irf7-R	CATTTCCTCCACTTGGCTGAG	
epc rig-i-F	TGCTGGACCGGATGTGTTATCT	
epc rig-i-R	TGGTGATCGATGGTTCGATTCT	
epc cyp19a1a-F	CTAGGAGTCCCTCTCAATGAGCGT	
epc cyp19a1a-R	GGCATCCTGTAACTCCTGAGCTTC	
epc atg14-F	TGGAAGAAACCCGGAGAGGTATTC	
epc atg14-R	CGCAACAACAGCTCCTTGCCACTC	
epc β-actin-F	CACTGTGCCCATCTACGAG	
epc β-actin-R	CCATCTCCTGCTCGAAGT	
caIFN-A-F	GCTTCGGGAAATGAGTGGACAAT	
caIFN-A-R	TTCACTTTTGTTAGATTCCATTGCG	

caIFN-B-F	GGACACCTGTACAACTTGATTGGAG	
caIFN-B-R	GCTCTGGATTCATGTGTTCTTTGG	
caβ-actin-F	AGACCAGCAAATACTATGTCACCA	
caβ-actin-R	GGCACAGTTCCAATACCTCCAA	
SVCV-g-F	CGACCTGGATTAGACTTG	
SVCV-g-R	AATGTTCCGTTTCTCACT	
SVCV-l-F	GCCCACTTTGCATCCAGTCC	
SVCV-l-R	GAGATGCCACAGACTCCTCC	
SVCV-m-F	TACTCCTCCCACTTACGA	
SVCV-m-R	CAAGAGTCCGAGAAGGTC	
SVCV-n-F	TGAGTGCTGAGGACGAT	
SVCV-n-R	TTTGTGAGTTGCCGTTA	
SVCV-p-F	TTGGACCTGGGATAGTGA	
SVCV-p-R	CTTGCTTGGTTTGTGGG	
