# Supplementary figures and images for: Fish female-biased gene cyp19a1a leads to female antiviral response attenuation between sexes by autophagic degradation of MITA
Source: PLoS Pathog. 2022 Jun 21;18(6):e1010626. doi: 10.1371/journal.ppat.1010626 (PMC9249237; doi:10.1371/journal.ppat.1010626)

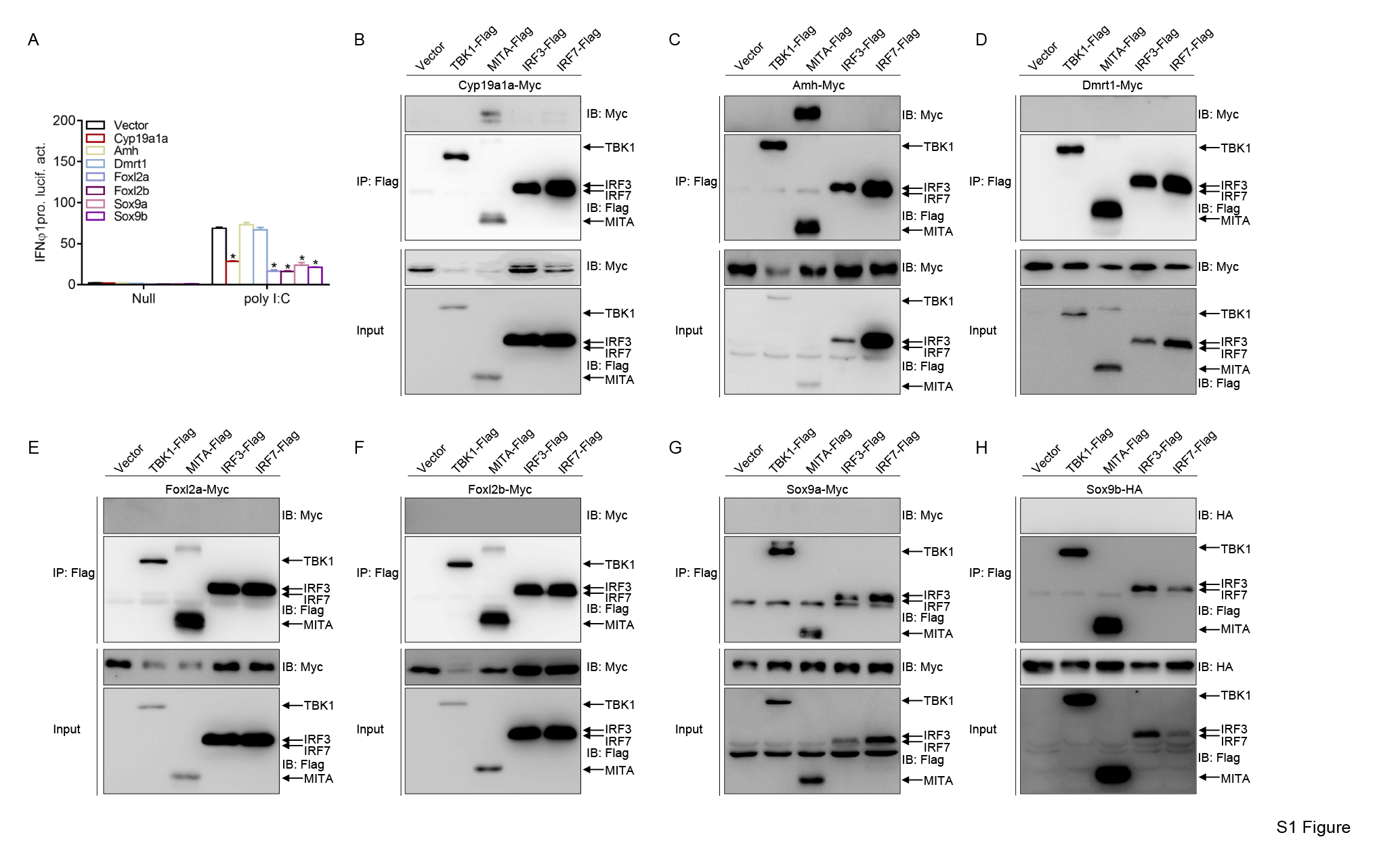

Supplement: S1 Fig — (A) Transfected with 250 ng IFNφ1pro and 25 ng pRL-TK, plus 250 ng Cyp19a1a-Myc/Amh-Myc/Dmrt1-Myc/Foxl2a-Myc/Foxl2b-Myc/Sox9a-Myc/Sox9b-Myc or pCMV-Myc (control vector). After 24 h, cells were transfected with poly I:C (1 μg). Luciferase activities were monitored. The promoter activity is presented as relative light units (RLU) normalized to Renilla luciferase activity. (B-H) EPC cells were transfected with the indicated plasmids (5 μg each). After 24 h, cell lysates were IP with anti-Flag affinity gel. Then the immunoprecipitates and WCLs were analyzed by IB with the anti-Myc or anti-HA, and anti-Flag Abs, respectively. (TIF) [file ppat.1010626.s002.tif]
